# Addendum and Errata

**Published:** 1880-02

**Authors:** 


ADDENDUM.—Our esteemed contemporary, the *London Practitioner*, is apprehensive that our name, THE PRACTITIONER, without some qualification, might be calculated to confuse or mislead some of its American subscribers, and has written politely requesting us to make such addition to it as would be likely to obviate any confusion in the matter. This we cheerfully do, and instead of on our title page, "THE PRACTITIONER: An *Independent* Monthly Journal," etc., *transpose* the word *Independent*, and it will henceforth appear under the title of "THE INDEPENDENT PRACTITIONER: A Monthly Journal," etc. We trust that this change will be entirely satisfactory to the *eldest* of the PRACTITIONER *family*. There are several members of the *name* on this side of the water, and as we desire to be on the most cordial, fraternal relations with *all* of them, we trust that they, too, will kindly receive us into the brotherhood of which they are members.

ERRATA.—We regret that, in our January issue, the printer neglected to give credit to the *Medical and Surgical Brief*, N. Y., for the article entitled "A Pleasant Remedy for Toothache," by T. C. Osborn, M. D., Greensboro, Ala.

Page 26, Article VI., last line, for the word "we" read "they."

Page 100, second paragraph, last line, "10,000" read "100,000."

